# Evaluation of a continuous blood glucose monitoring system using a central venous catheter with an integrated microdialysis function

**DOI:** 10.1186/cc10777

**Published:** 2012-03-20

**Authors:** F Möller, J Liska, A Öwall, A Franco-Cereceda

**Affiliations:** 1 Karolinska Institutet, Solna, Sweden

## Introduction

Glycemic control in critically ill patients has been debated over the last decade. An accurate glucose monitoring system is essential to understand and study this concern. We have evaluated the accuracy and technical feasibility of a continuous glucose monitoring system using intravascular microdialysis.

## Methods

Thirty patients undergoing cardiac surgery were monitored using a triple-lumen central venous catheter (Eirus TLC^®^; Dipylon Medical AB, Sweden) with an integrated microdialysis membrane. The catheter was placed with the tip in the superior vena cava, and functions both as a central venous catheter, enabling blood sampling and administration of medication, while simultaneously measuring glucose. The patients were monitored for up to 48 hours postoperatively in the ICU. As reference, arterial blood samples were taken every hour.

## Results

**Figure 1:**
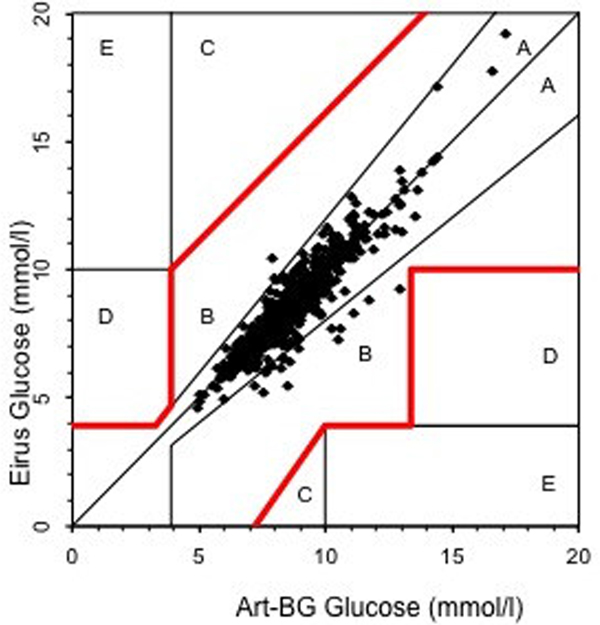
**Clarke error grid analysis**.

**Figure 2:**
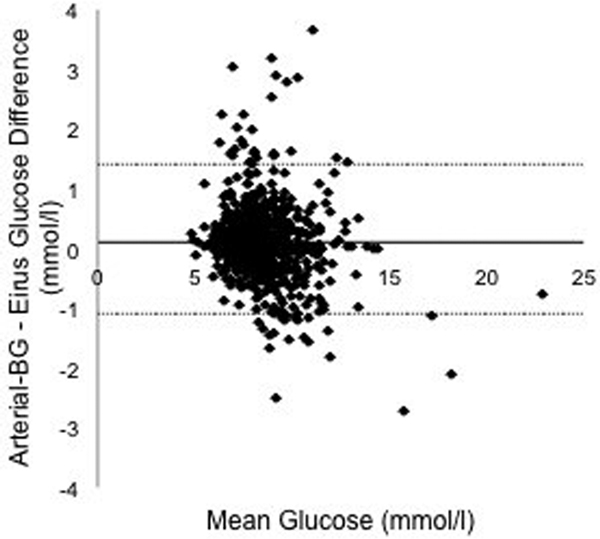
**Bland-Altman analysis**.

Data were available from all 30 patients. A total of 725 paired (arterial blood gas-microdialysis) samples were obtained. Glucose correlation coefficient was 0.87. Using Clarke error grid analysis, 100% of the paired samples were in region AB and 97.4% in region A (Figure 1). Mean glucose level was 8.6 mmol/l, bias -1.3% and mean absolute relative difference was 4.8%. A total 97.5% of the paired samples were correct according to ISO criteria. Bland-Altman analysis showed bias ± limits of agreement -0.11 ± 1.3 mmol/l (Figure 2).

## Conclusion

Central venous microdialysis is an accurate and reliable method for continuous blood glucose monitoring in critically patients.

